# Comparison of Microstructure and Mechanical Properties of Ultra-Narrow Gap-Welded and Submerged Arc-Welded Q355E HSLA Steel

**DOI:** 10.3390/ma18122805

**Published:** 2025-06-14

**Authors:** Youqi Wang, Renge Li, Qingnian Wen, Wenkai Xiao, Shang Wu, Xian Zhai, Fuju Zhang

**Affiliations:** 1 School of Materials Science and Engineering, Dalian Jiaotong University, Dalian 116028, China; 19818962576@163.com; 2 China Construction Science and Industry Corporation, Ltd., Wuhan 430072, China; lirenge@cscec.com (R.L.); wenqingnian@cscec.com (Q.W.); 3 School of Power and Mechanical Engineering, Wuhan University, Wuhan 430072, China; 2018302080322@whu.edu.cn (S.W.); zhaixian@whu.edu.cn (X.Z.); 4 Wuhan Narogap Intelligent Welding Equipment Co., Ltd., Wuhan 430072, China; fjzhang@whu.edu.cn

**Keywords:** HSLA steel thick plates, UNGW, acicular ferrite, microstructure, mechanical properties

## Abstract

Reasonable welding methods are of great significance for optimizing the microstructure and ensuring the mechanical properties of welded joints. In this study, ultra-narrow gap welding (UNGW) and submerged arc welding (SAW) were employed to weld Q355E high-strength low-alloy (HSLA) steel thick plates, and the microstructure and mechanical properties of the welded joints were systematically characterized. The UNGW welded joint exhibits superior comprehensive mechanical properties: a room-temperature tensile strength of 664 MPa with 43.1% elongation at fracture, along with higher microhardness and enhanced impact performance at −40 °C, all of which significantly outperform SAW welded joints. This advantage primarily stems from the faster cooling rate during UNGW, which promotes the formation of beneficial acicular ferrite in the joint microstructure. This study provides theoretical support and technical guidance for welding HSLA steel thick plates.

## 1. Introduction

High-strength low-alloy (HSLA) steel is an engineering structural steel developed as early as the last century based on carbon steel, with a carbon content not exceeding 0.3 wt.%, a total alloying element content below 5 wt.%, and a yield strength no less than 375 MPa [1,2]. Its high strength primarily stems from solid solution strengthening by alloying elements [3,4], precipitation strengthening induced by thermomechanical processing, such as the precipitation of C- and N-based compounds and Cu-rich nanoscale phases [5,6], as well as grain refinement strengthening and transformation strengthening from phases like martensite and bainite. HSLA steel exhibits excellent comprehensive mechanical properties, good weldability, remarkable corrosion resistance, and relatively low cost, making it widely used as an important type of structural plate in engineering machinery for various fields, including petrochemical, aerospace, power, bridge, and shipbuilding industries [7,8,9,10,11].

With the extensive applications, a large number of welded structures exist in HSLA steel components, and the quality of welding directly affects their service performance and safety reliability. Therefore, while developing HSLA steel, it is equally important to research welding processes that match its performance and are more environmentally friendly and efficient [12,13,14], especially for the welding of thick HSLA steel plates, which presents greater practicality and challenges. The heat input in welding is calculated by the formula Q = ηUI/v, where Q is the heat input (J/cm), η is the thermal efficiency (which varies slightly depending on the welding method), U is the welding voltage (V), I is the welding current (A), and v is the welding speed (cm/s). This demonstrates that the welding heat input is directly proportional to the welding voltage and current. Ultra-narrow gap welding (UNGW), as a new welding technology developed from narrow gap welding methods, features lower welding voltage and current, meaning it has smaller heat input. This, combined with UNGW’s narrower groove configuration, results in a narrower heat-affected zone (HAZ) in the welded joint, aiming to achieve high-performance welded joints [15,16,17]. However, in UNGW, since the weld is almost perpendicular to the welding direction, poor sidewall fusion often occurs [18,19,20]. Ensuring sidewall fusion, improving weld formation efficiency, and preventing weld cracks remain critical issues to be addressed in UNGW.

In HSLA steels, the microstructural evolution of welded joints significantly influences their mechanical properties. Research indicates that the coarse-grained heat-affected zone (HAZ) of welded joints forms non-uniform microstructures after welding, such as retained δ-ferrite, residual γ-austenite, and martensite, which severely determine the mechanical properties of the welded joints [21,22]. Acicular ferrite (AF), a typical microstructure formed during austenite-to-ferrite transformation, nucleates heterogeneously on non-metallic inclusions within austenite grains and grows through compositional diffusion [23,24]. Multi-phase microstructures dominated by AF or containing AF as the primary phase exhibit excellent comprehensive properties in HSLA steels [25,26,27]. The formation of AF is closely related to austenitization conditions and continuous cooling rates. Studies have shown that high cooling rates suppress the formation of polygonal ferrite and pearlite while promoting the transformation to AF and bainite [28,29]. Therefore, increasing the cooling rate during welding to facilitate AF formation and optimizing microstructures to enhance the overall mechanical properties of welded joints are of great significance.

In our previous study [30], the multi-functional integrated ultra-narrow gap gas metal arc welding developed by Zhang et al. was employed to weld Q235 low-carbon steel thick plates, achieving excellent sidewall fusion and superior mechanical properties in the welded joints. Notably, the AF in the welded joints played a crucial role in performance regulation. To investigate the applicability of the UNGW process for thick plates of HSLA steel and to expand its potential industrial applications, in this study, Q355E HSLA steel thick plates were welded using UNGW and submerged arc welding (SAW), respectively. The microstructural characteristics and mechanical properties of the two welded joints were systematically compared to provide theoretical guidance for welding HSLA steel thick plates.

## 2. Experimental Section

### 2.1. Welding Processes

**Figure 1:**
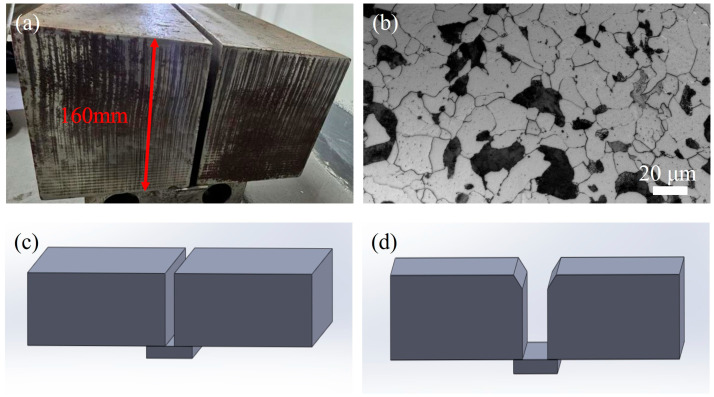
(**a**) Photograph of Q355E thick plate; (**b**) OM image of Q355E thick plate; (**c**) square groove of UNGW, and (**d**) root with U-groove and face with V-groove of SAW.

The base material (BM) employed in this study was Q355E HSLA steel thick plate with dimensions of 300 L × 150 W × 160 H mm, as shown in Figure 1a. The optical microscopy image of the BM (Figure 1b) revealed a typical ferrite plus pearlite structure. Two Q355E HSLA steel plates were welded using ultra-narrow gap gas metal arc welding (hereinafter referred to as UNGW) and submerged arc welding (SAW), respectively. The UNGW process employed a square groove configuration (Figure 1c), while the SAW process used a combined U-V groove design with a U-shaped root and V-shaped top section (Figure 1d). The chemical compositions of the Q355E BM and the welding wires for both processes are listed in Table 1. The welding parameters for both processes are presented in Table 2, showing that UNGW exhibited significantly lower welding power compared to SAW.

### 2.2. Microstructure Characterization

Specimens were extracted from the weld zone (WZ), heat-affected zone (HAZ), and BM of both welded joints. The specimens were sequentially ground using 400–2000 grit sandpaper, followed by mechanical polishing with 2.5 μm, 0.5 μm, and 20 nm SiO_2_ (OPS polishing paste). The polished specimens were etched by swabbing 4–6 times with 4% nital solution, and the microstructure of the welded joints was then observed at low magnification using optical microscopy (OM) equipment (Carl Zeiss Axio Lab.A1, Jena, Germany). For microstructural characterization of different regions and fracture morphology after mechanical testing, scanning electron microscopy (SEM) equipment (TESCAN MIRA3 LMH, Brno, Czech Republic) equipped with both an energy dispersive spectrometer (EDS) (Aztec Energy Standard X-Max 20, Oxford Instruments, Abingdon, UK) and an electron backscatter diffraction (EBSD) detector (Aztec HKL) was used. Specimens for SEM and EBSD analysis (excluding fracture specimens) underwent identical polishing procedures to those of the OM specimens but without the etching step.

### 2.3. Mechanical Properties Testing

**Figure 2:**
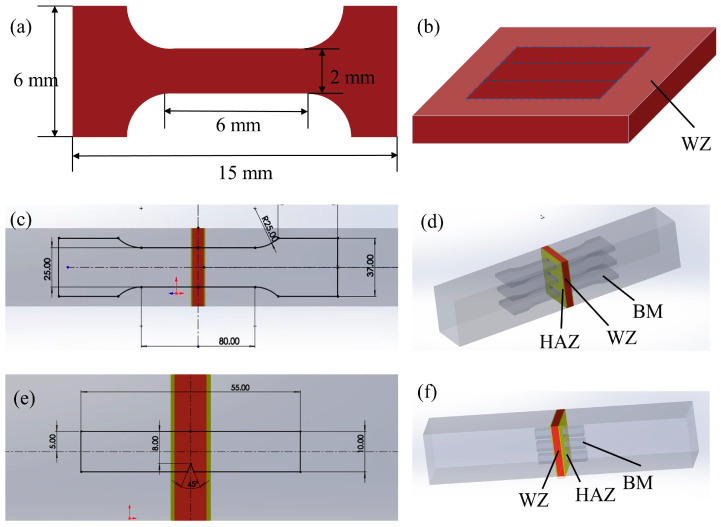
(**a**,**b**) Schematic diagram of small-sized tensile specimens and sampling locations within the WZ. (**c**,**d**) Schematic diagram of large-sized tensile specimens with reference to GB/T 2651-2023 of the Chinese National Standard [31] and sampling locations for the entire welded joint, and (**e**,**f**) schematic diagram of low-temperature impact specimens with reference to GB/T 2650-2022 [32] and sampling locations for the entire welded joint.

The microhardness across different regions of the welded joints was measured using an HVS-1000A microhardness tester, with a total of 100 measurement points taken at 0.25 mm intervals from the WZ to the BM. Each specimen underwent 100 indentations under a 500 g load with a 10 s dwell time. Small-scale tensile testing of the WZ for both welded joints was performed at room temperature using an MTS E45.105 universal testing machine (MTS Systems, Eden Prairie, MN, USA), with a gauge length of 6 mm and a crosshead speed of 0.5 mm/min. Full-size tensile testing of the complete welded joints was conducted on the same MTS E45.105 machine, employing an 80 mm gauge length and 5 mm/min testing speed. Charpy impact tests at −40 °C were carried out on complete welded joints using a JB-30B impact tester, with 55 mm long specimens cooled in alcohol within a low-temperature thermostat chamber. The schematic diagrams and corresponding sampling locations of small-scale tensile tests, large-scale tensile tests, and Charpy impact tests at −40 °C are shown in Figure 2. Each test was conducted with three repeated trials.

## 3. Results

### 3.1. Microstructure

**Figure 3:**
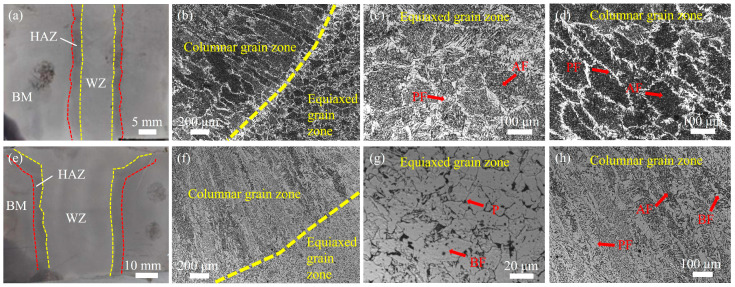
(**a**) Photograph of a UNGW weld joint; (**b**–**d**) OM images of the WZ in the UNGW welded joint; (**e**) photograph of an SAW weld joint, and (**f**–**h**) OM images of the WZ in the SAW welded joint. PF: proeutectoid ferrite; AF: acicular ferrite; P: pearlite; BF: blocky ferrite.

Figure 3 presents macrographs of welded joints and microstructural images of the WZ under both welding processes. Comparative analysis of Figure 3a,e reveals that the UNGW joint exhibits a significantly narrower WZ and HAZ. Figure 3b shows the fusion line between two adjacent weld passes in the UNGW joint, where the prior austenite grains above the fusion line clearly display characteristic columnar crystals of as-cast microstructure, while equiaxed grains are observed below. These columnar grains, formed at the root of each weld pass, result from austenite growth along the maximum temperature gradient direction (perpendicular to the fusion line) during weld metal solidification. The equiaxed zone, located between the surface region of the preceding pass and the root region of the subsequent pass, forms due to remelting of the previous pass under a more uniform temperature distribution during subsequent welding. In the WZ under UNGW, both the equiaxed zone (Figure 3c) and columnar grain zone (Figure 3d) exhibit similar microstructural characteristics, consisting of coarse white proeutectoid ferrite (PF) along the grain boundaries and finer interlocking AF within the grains. The preferential formation of PF at the grain boundaries is attributed to their higher energy state. In contrast, the WZ equiaxed zone under SAW (Figure 3g) comprises blocky ferrite (BF) and pearlite (P), where ferrite from the previous pass undergoes repeated austenitization during subsequent thermal cycles without complete cooling. The uniform temperature field produces randomly oriented austenite grains, while slow ferrite nucleation under low undercooling prevents significant PF formation at the grain boundaries. The SAW columnar grain zone (Figure 3h) features elongated, coarse prior austenite grains with PF along boundaries and mixed blocky/acicular ferrite within the grains. The high heat input in SAW extends the dwell time above Ac3 temperature, promoting substantial austenite grain growth. During cooling, PF preferentially nucleates at the grain boundaries, followed by progressive intragranular ferrite formation with decreasing temperature.

**Figure 4:**
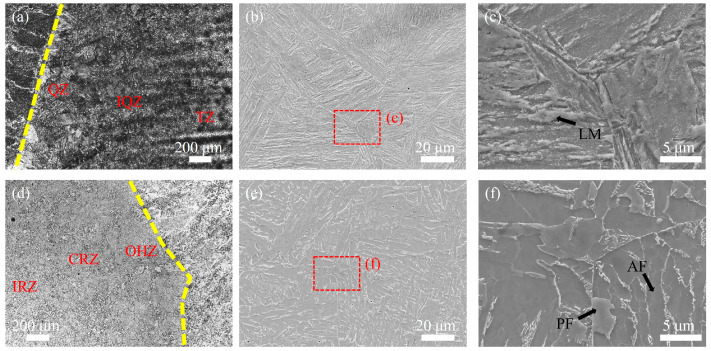
(**a**) Microstructure of the HAZ: (**a**) OM image of UNGW; (**b**,**c**) SEM image of the quenching zone in the UNGW welded joint; (**d**) OM image of SAW, and (**e**,**f**) SEM images of the overheated zone in the SAW welded joint. QZ: quenching zone; IQZ: incomplete quenching zone; TZ: transition zone; OHZ: overheated zone; CRZ: complete recrystallization zone; IRZ: incomplete recrystallization zone.

Figure 4 displays microstructural images of the HAZ for both welding methods. Figure 4a shows the HAZ microstructure of the UNGW joint, which can be divided into three distinct regions: the quenched zone (QZ) near the fusion line, the intermediate incomplete quenched zone (IQZ), and the transition zone (TZ) adjacent to the BM. The QZ, located closest to the fusion line, represents the partially melted region where temperatures exceeded the solidus line during welding. In this area, the original base metal and weld metal structures were completely austenitized, resulting in significantly coarsened austenite grains. Due to the low heat input characteristic of UNGW and the consequent rapid cooling rate, these austenite grains transformed into lath martensite (LM), as clearly shown in Figure 4b,c. Figure 4d presents the HAZ image of the SAW joint. The IQZ exhibits finer grain size, having experienced peak temperatures between Ac3 and 1100 °C during welding. In this temperature range, the original ferrite and pearlite in the base metal completely transformed into austenite, but the relatively lower temperatures (compared to the QZ) limited grain growth, resulting in finer austenite grains. The TZ, experiencing temperatures between Ac1 and Ac3, underwent only partial austenitization of the ferrite–pearlite structure. The limited time at these lower temperatures restricted grain growth, yielding fine ferrite grains upon cooling. This zone also shows banded dark carbide precipitates formed due to carbon rejection during cooling, since austenite has higher carbon solubility than ferrite. Some untransformed regions retained the original base metal ferrite–pearlite structure. The HAZ under SAW (Figure 4d) shows no quenching effects and consists of three distinct regions: the overheated zone (OHZ) near the fusion line, a complete recrystallization zone (CRZ), and an incomplete recrystallization zone (IRZ) adjacent to the BM. The OHZ microstructure consists of network-like PF along the grain boundaries and AF within the grains, formed during austenite decomposition. The CRZ contains fine ferrite and pearlite resulting from complete austenitization followed by cooling. The IRZ, having undergone only partial austenitization, shows a mixture of transformed ferrite–pearlite and slightly coarsened untransformed structures, resulting in non-uniform grain size distribution.

### 3.2. Mechanical Properties

**Figure 5:**
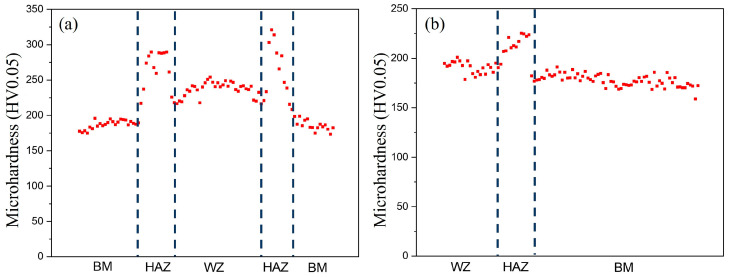
Microhardness of the two weld joints: (**a**) UNGW and (**b**) SAW.

Figure 5 presents the microhardness test results for both welded joints. The BM region of Q355E steel exhibited consistent hardness values (~185 HV0.5) in both joints. Both welding methods showed identical hardness distribution patterns across the three characteristic zones: HAZ > WZ > BM. In the UNGW joint (Figure 5a), the peak hardness of ~290 HV0.5 occurred in the HAZ, while the WZ showed a hardness of ~250 HV0.5. By contrast, the SAW joint (Figure 5b) demonstrated significantly lower hardness values, with the HAZ and WZ being ~225 HV0.5 and ~200 HV0.5, respectively—both markedly softer than their UNGW counterparts.

**Figure 6:**
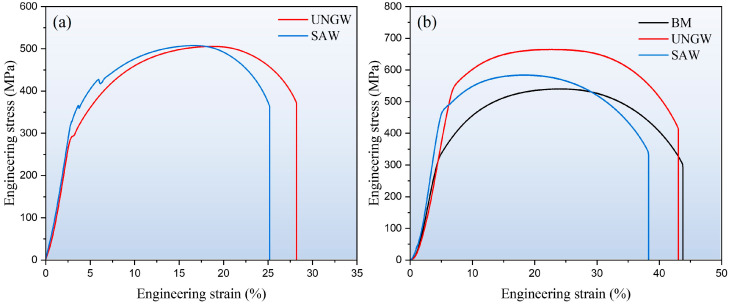
(**a**) The large-scale tensile engineering stress–strain curves of whole welded joints, and (**b**) the small-scale tensile engineering stress–strain curves of the WZ.

Figure 6a presents the engineering stress–strain curves from full-scale tensile tests of both welded joints. Since these tests incorporated all three zones (WZ, HAZ, BM), the resulting curves reflect the overall joint performance, with strength being determined by the weakest region. As indicated by the hardness distribution in Figure 5, the BM represented the softest zone. Both joints exhibited identical tensile strengths (~510 MPa), corresponding to the intrinsic strength of the Q355E BM. Notably, the UNGW joint showed approximately 3% higher fracture elongation. To evaluate the actual strength of the weld zones, reduced-section tensile tests were conducted on specimens extracted from the WZ and BM of both joints (Figure 6b, Table 3). The BM displayed the lowest ultimate tensile strength (540 MPa), consistent with the full-scale test results in Figure 6a. The UNGW WZ specimens demonstrated superior mechanical properties, including the highest yield strength (545 MPa), ultimate tensile strength (664 MPa), and ductility comparable to the BM (43.1% elongation). In contrast, while the SAW WZ specimens showed improved strength over the BM, their elongation was slightly reduced (38.3%). Overall, the UNGW joint exhibited the best combination of strength and ductility.

**Figure 7:**
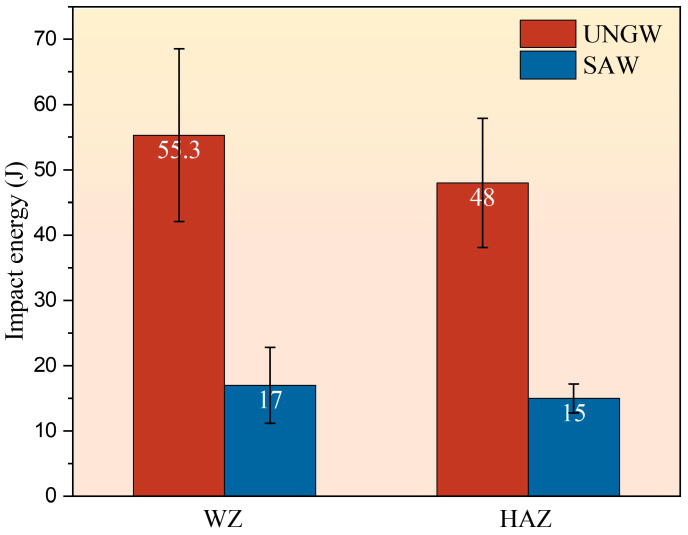
The impact energy of specimens from the WZ and HAZ in UNGW and SAW welded joints at −40 °C.

Figure 7 presents the Charpy impact test results of both welded joints at −40 °C for WZ and HAZ regions. The UNGW joint exhibited average impact absorbed energies of approximately 55 J for the WZ and 48 J for the HAZ. In comparison, the SAW joint showed significantly lower values, with average impact energies of 17 J for the WZ and 15 J for the HAZ. These results clearly demonstrate that the UNGW joint possesses superior low-temperature impact toughness in both WZ and HAZ regions compared to the SAW joint.

## 4. Discussion

### 4.1. Strengthening and Toughening Mechanisms

**Figure 8:**
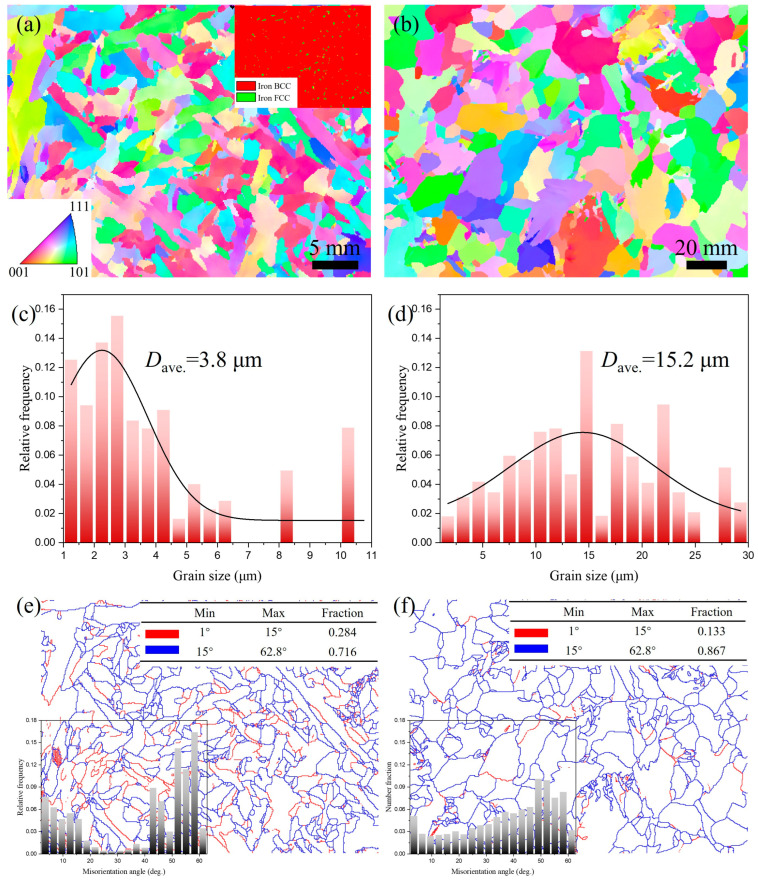
EBSD-IPF map of the WZ: (**a**) UNGW, and the inset is the relevant phase distribution map; (**b**) SAW. Grain size statistical graph: (**c**) UNGW; (**d**) SAW. Grain boundary distribution, and the inset is the misorientation angle distribution diagram: (**e**) UNGW, and (**f**) SAW.

Figure 8 presents EBSD-IPF maps of the WZ for both welded joints, along with corresponding grain size statistics and grain boundary distribution maps. The WZ of the UNGW joint (Figure 8a) contains numerous slender AF grains with retained FCC austenite and relatively larger PF precipitates at the grain boundaries. In contrast, the SAW joint (Figure 8b) primarily consists of coarser BF. The average grain sizes in the WZ were measured as 3.8 μm for UNGW and 15.2 μm for SAW, demonstrating significantly finer microstructure in UNGW. This refinement primarily results from AF nucleation during austenite decomposition, where AF effectively subdivides prior austenite grains into smaller domains that subsequently accommodate further ferrite nucleation [33]. According to the Hall–Petch relationship [34], the grain refinement contribution to matrix strength can be expressed as

$$\Delta \sigma_{M-G}=KD^{-1/2}$$

where K is a constant with a value of approximately 0.21 MPa∙m^1/2^ [35], and *D* represents the average grain size. Calculations show that the grain boundary strengthening contributions for the WZ of UNGW and SAW joints are 108 MPa and 54 MPa, respectively. This demonstrates that grain boundary strengthening in the UNGW joint provides approximately twice the strengthening effect compared to the SAW joint.

Figure 8e,f present the corresponding grain boundary distribution maps and misorientation angle distribution diagram for Figure 8a,b. The low-angle grain boundary (LAGB, 1–15°) fractions in UNGW and SAW specimens are 0.284 and 0.133, respectively. This difference primarily results from the presence of finer AF grains in UNGW, which introduce high-density dislocations and more substructures, thereby increasing the LAGB proportion. Furthermore, the misorientation angle distribution in UNGW exhibits a characteristic bimodal pattern (with both low-angle and high-angle peaks), while SAW shows a relatively uniform distribution. In UNGW specimens, the low-angle misorientations originate from substructures and dislocations, whereas the high-angle misorientations arise from the large orientation differences between adjacent AF grains. These analyses confirm that dislocation strengthening plays a more significant role in UNGW specimens compared to SAW.

**Figure 9:**
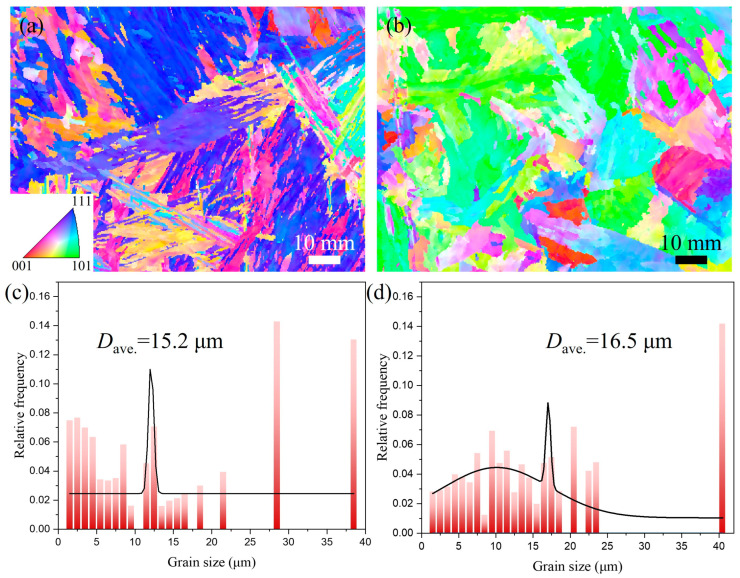
EBSD-IPF map of HAZ: (**a**) UNGW; (**b**) SAW, and corresponding grain size statistical graph: (**c**) UNGW, and (**d**) SAW.

Figure 9 presents EBSD-IPF maps and corresponding grain size statistics for the QZ in the UNGW joint and the OHZ in the SAW joint. These regions underwent phase transformations during welding, exhibiting significantly different microstructures compared to the base metal, with their morphological characteristics playing crucial roles in determining joint mechanical properties. As shown in Figure 9a, the QZ of the UNGW joint primarily consists of LM. Notably, no FCC-structured retained austenite was detected in this zone, indicating the absence of detrimental M-A constituents [36] that could deteriorate HAZ mechanical properties. The LM, formed through non-equilibrium phase transformation, contains carbon in solid solution due to insufficient time for carbide precipitation during rapid cooling, resulting in substantial solid solution strengthening and consequently the significant hardness increase observed in the HAZ (Figure 5a). In contrast, the OHZ of the SAW joint (Figure 9b) experienced complete austenitization during welding, leading to the formation of coarse network-like PF along prior austenite grain boundaries—a microstructure unfavorable for mechanical performance. Grain size statistics (Figure 9c,d) reveal average grain sizes of 15.2 μm and 16.5 μm for UNGW and SAW, respectively, with UNGW showing a slightly finer microstructure.

The EBSD characterization of both welded joints demonstrates that the UNGW joint exhibits finer grain sizes in both the WZ and HAZ, with particularly significant grain boundary strengthening effects in the WZ. Moreover, the UNGW joint contains more beneficial components, abundant AF in the WZ and LM in the HAZ, which contribute to dislocation strengthening and transformation strengthening. It is through these combined strengthening mechanisms that the UNGW joint achieves superior comprehensive mechanical properties compared to the SAW joint.

**Figure 10:**
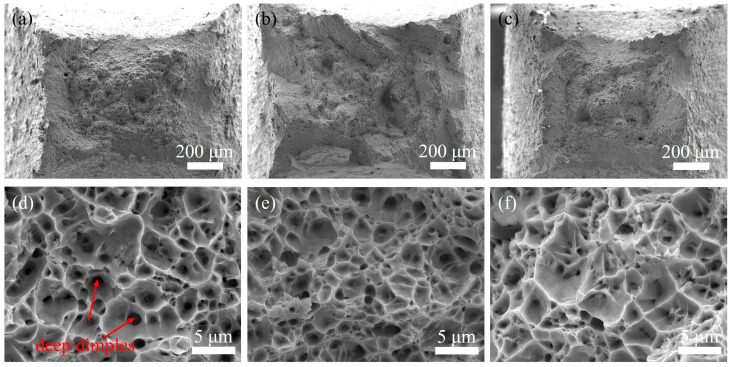
Fracture surface of small-scale tensile specimens: (**a**,**d**) WZ of UNGW; (**b**,**e**) WZ of SAW, and (**c**,**f**) BM.

Figure 10 shows the fracture morphologies of reduced-section tensile specimens from both weld zones (UNGW and SAW) and Q355E HSLA steel BM. All three fracture surfaces exhibit numerous dense dimples, characteristic of ductile fracture. Comparative analysis reveals that the SAW specimen shows a slightly lower reduction of area, consistent with its relatively lower fracture elongation in the tensile curve (Figure 6b). Furthermore, both UNGW and BM specimens display larger dimple sizes, with the UNGW specimen particularly showing a higher proportion of deep dimples featuring superior plasticity.

**Figure 11:**
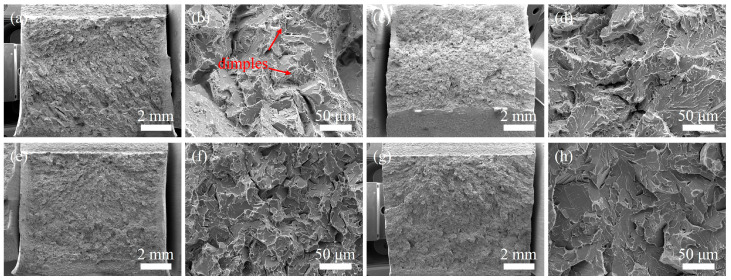
Fracture surface of impact test specimen: (**a**,**b**) WZ of UNGW; (**c**,**d**) HAZ of UNGW; (**e**,**f**) WZ of SAW, and (**g**,**h**) HAZ of SAW.

Figure 11 presents the fracture morphologies corresponding to the −40 °C impact tests shown in Figure 7. The impact fracture surface of the WZ under UNGW exhibits mixed-mode characteristics combining cleavage fracture and dimple rupture (Figure 11a,b), indicating that while brittle fracture was initiated during low-temperature impact testing, the material retained ductile fracture features. In contrast, the fracture surface of the WZ under SAW shows significantly fewer and smaller dimples (Figure 11e,f), demonstrating its inferior low-temperature impact toughness compared to the UNGW joint. Comparative analysis of the HAZ fracture surfaces reveals that the UNGW specimens primarily underwent cleavage fracture with limited small dimples, while the SAW specimens exhibited pure shear fracture with flat surfaces, displaying more pronounced brittle fracture characteristics.

The WZ of the UNGW joint mainly contains PF at the grain boundary and AF inside. These network PF instances at the grain boundary are usually weak points where cleavage cracks easily initiate and propagate, causing brittle fracture. In contrast, AF has higher stability with large misorientation angles between adjacent AF and an interlocking growth mechanism. The small grain size of AF blocks crack propagation, requiring higher energy for crack deflection, giving the UNGW WZ higher impact toughness. The HAZ of the UNGW joint is mainly LM with some toughness, but it is less effective than AF, thus showing limited dimples. The better low-T impact performance of the UNGW joint comes from its AF and LM, mainly due to lower heat input during welding.

### 4.2. AF Introduced in UNGW

**Figure 12:**
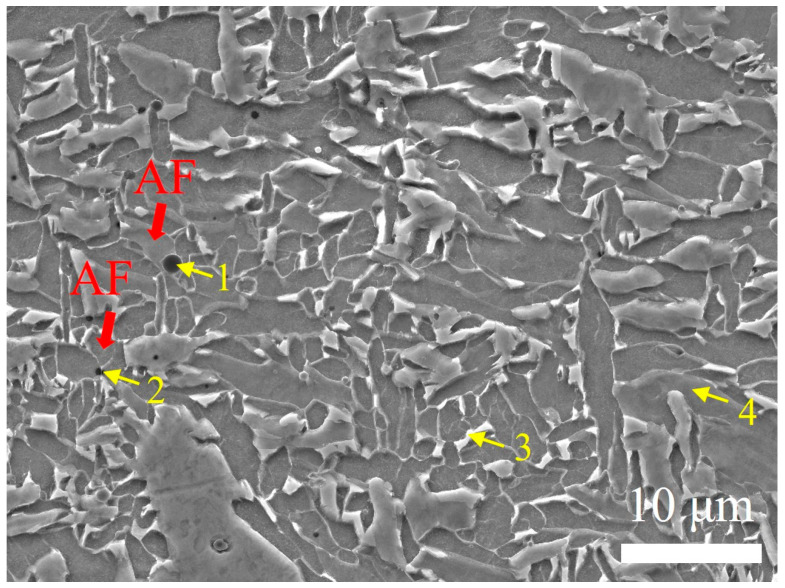
SEM image of the WZ in the UNGW welded joint.

Figure 12 shows the SEM morphology of the WZ in the UNGW joint, revealing numerous black particulate impurities with substantial AF formation around them. EDS analysis was conducted at four locations marked in the figure, with results summarized in Table 4. Compared to brighter regions (Point 3) and darker matrix areas (Point 4), the black impurities (Points 1–2) exhibit high concentrations of O, Mn, Si, and Al—particularly elevated O content, indicating oxide precipitation of these elements. Notably, these impurity sites effectively promote intragranular AF nucleation in their vicinity. This impurity-induced AF formation phenomenon has been previously reported in the literature [37].

In this study, UNGW exhibited lower heat input and faster cooling rates than SAW. Wang et al. [38] investigated the effect of cooling rate on AF nucleation in 35MnVS steel. The results showed that as the cooling rate increased, the size of TiN-MnS inclusions decreased while their number increased. When the cooling rate increased from 0.2 °C/s to 10 °C/s, the average inclusion size decreased from 2.7 µm to 1.3 µm. With an increasing cooling rate, the austenite grain size decreased, and the grain size distribution became more uniform. The AF content first increased and then decreased, reaching a maximum of 82.6% at a cooling rate of 5 °C/s. These findings demonstrate the influence of cooling rate on inclusion characteristics and AF formation. In this study, the higher cooling rate in UNGW effectively promoted AF nucleation and uniformity, significantly improving the mechanical properties of the welded joint.

## 5. Conclusions

In this study, Q355E high-strength low-alloy steel thick plates were welded using ultra-narrow gap gas metal arc welding (UNGW) and submerged arc welding (SAW) processes, respectively. The microstructure, mechanical properties, and strengthening–toughening mechanisms of the two welded joints were studied. The main conclusions are as follows.

(1) The UNGW joint exhibited narrower weld and heat-affected zones. Both UNGW and SAW joints showed distinct columnar grain and equiaxed grain zones in the weld zone, while the microstructures of these two zones differ between the two welded joints. In UNGW joints, abundant acicular ferrite was found within the grains in both columnar and equiaxed zones, and network proeutectoid ferrite precipitated along prior austenite grain boundaries. In contrast, the SAW columnar zone showed similar characteristics to UNGW but with less acicular ferrite and more blocky ferrite, while its equiaxed zone mainly consisted of pearlite and blocky ferrite. Additionally, the heat-affected zone of the UNGW joint experienced quenching effects, being divided into a quenched zone, an incomplete quenched zone, and a transition zone, in which lath martensite with good performance was identified. However, the SAW heat-affected zone showed no quenching effects and consisted of overheated, complete recrystallization and incomplete recrystallization zones.
(2) The UNGW welded joint exhibits superior comprehensive mechanical properties compared to the SAW welded joint. Specifically, the microhardness of the weld and heat-affected zones in the UNGW joint reached 290 HV0.5 and 250 HV0.5, respectively. The room-temperature tensile strength of the weld zone reached 664 MPa, which is 124 MPa higher than that for the base material and 80 MPa higher than that for the SAW weld zone, while maintaining a comparable fracture elongation of 43.1%. Additionally, the impact toughness of the weld and heat-affected zones at −40 °C was 55 J and 48 J, respectively, significantly exceeding the 17 J and 15 J of the SAW specimens.
(3) The outstanding mechanical properties of the UNGW welded joint primarily result from the formation of a large amount of acicular ferrite in the welded joint, which contributes to notable grain boundary strengthening, dislocation strengthening, and phase transformation strengthening. The formation of acicular ferrite is closely related to the cooling rate during welding and the size of precipitated inclusions in the joint structure. These factors are particularly attributed to the lower heat input and faster cooling rate characteristic of the UNGW process.

In conclusion, UNGW demonstrates unique advantages in the welding of high-strength low-alloy steel thick plates, which suggests tremendous application potential in shipbuilding, bridge construction, and energy transmission systems. Based on these findings, further investigations into the corrosion resistance and other comprehensive properties of UNGW-welded high-strength low-alloy steel joints, along with optimization of welding process parameters, represent important research directions.

## Figures and Tables

**Table 1:** Chemical composition of Q355E high-strength and low-alloy steel, and corresponding welding wire of ultra-narrow gap welding (UNGW) and submerged arc welding (SAW), respectively.

Element [wt.%]	C	Mn	Si	S	P	Cr	Ni	Ti	Cu	Mo	V	Fe
Q355E	≤0.15	≤0.15	≤0.5	≤0.02	≤0.025	/	≤0.5	≤0.2	/	/	≤0.15	Bal.
UNGW	0.06–0.15	1.40–1.95	0.80–1.15	≤0.025	≤0.025	≤0.15	≤0.15	/	≤0.50	≤0.15	≤0.03	Bal.
SAW	≤0.12	≤0.07	≤0.015	≤0.015	≤0.015	≤0.20	≤0.30	/	≤0.35	/	/	Bal.

**Table 2:** Welding parameters for the UNGW and SAW.

Process	Welding Current [A]	Welding Voltage [V]	Welding Speed [mm/s]
UNGW	260–270	28–30	4.0–4.5
SAW	650–750	32–34	5.6–10

**Table 3:** Large-scale tensile properties of whole welded joints, and small-scale tensile properties of the BM and the weld zone from UNGW and SAW, respectively.

Testing	Process	Yield Strength [σ_YS_, MPa]	Ultimate Tensile Strength [σ_UTS_, MPa]	Elongation [δ, %]
large-scale tensile	UNGW	299.7 ± 6.5	497.4 ± 7.8	28.1 ± 0.9
	SAW	326.5 ± 35.9	488.5 ± 16.6	24.1 ± 3.4
small-scale tensile	BM	330.8 ± 15.5	537.7 ± 3.0	42.8 ± 2.2
	UNGW	570.8 ± 24.4	680.8 ± 14.6	38.2 ± 4.4
	SAW	445.4 ± 21.4	553.3 ± 26.9	39.1 ± 1.4

**Table 4:** The EDS point results of the WZ in the UNGW welded joint.

Element [wt.%]	C	O	Mn	Si	Ti	Al	Fe
1	1.76	16.66	9.06	7.20	0.32	5.46	Bal.
2	1.60	8.95	7.50	4.55	0.19	2.57	Bal.
3	6.52	/	1.26	0.71	/	/	Bal.
4	2.80	/	1.28	0.58	/	/	Bal.

## Data Availability

The original contributions presented in this study are included in the article. Further inquiries can be directed to the corresponding author.

## Nomenclature

HSLA	high-strength low-alloy	PF	proeutectoid ferrite
UNGW	ultra-narrow gap welding	BF	blocky ferrite
SAW	submerged arc welding	P	pearlite
HAZ	heat-affected zone	QZ	quenched zone
AF	acicular ferrite	IQZ	incomplete quenched zone
WZ	weld zone	TZ	transition zone
BM	base material	LM	lath martensite
OM	optical microscopy	OHZ	overheated zone
SEM	scanning electron microscopy	CRZ	complete recrystallization zone
EDS	energy dispersive spectrometer	IRZ	incomplete recrystallization zone
EBSD	electron backscatter diffraction	LAGBs	low-angle grain boundaries
