# Correction: Wang et al. Comparison of Microstructure and Mechanical Properties of Ultra-Narrow Gap-Welded and Submerged Arc-Welded Q355E HSLA Steel. *Materials* 2025, *18*, 2805

**DOI:** 10.3390/ma18204644

**Published:** 2025-10-10

**Authors:** Youqi Wang, Renge Li, Qingnian Wen, Wenkai Xiao, Shang Wu, Xian Zhai, Fuju Zhang

**Affiliations:** 1 School of Materials Science and Engineering, Dalian Jiaotong University, Dalian 116028, China; 19818962576@163.com; 2 China Construction Science and Industry Corporation, Ltd., Wuhan 430072, China; lirenge@cscec.com (R.L.); wenqingnian@cscec.com (Q.W.); 3 School of Power and Mechanical Engineering, Wuhan University, Wuhan 430072, China; 2018302080322@whu.edu.cn (S.W.); zhaixian@whu.edu.cn (X.Z.); 4 Wuhan Narogap Intelligent Welding Equipment Co., Ltd., Wuhan 430072, China; fjzhang@whu.edu.cn

In the published publication [1], there was an error regarding the affiliation for Youqi Wang. The corrected affiliation should be as follows: School of Materials Science and Engineering, Dalian Jiaotong University, Dalian 116028, China.

With this correction, the order of affiliations 1 and 3 has been adjusted accordingly.

The authors state that the scientific conclusions are unaffected. This correction was approved by the Academic Editor. The original publication has also been updated.
